# The Capability of a Novel All‐in‐One Sphincterotome With High Rotation and a Freely Bendable Blade for Various Endoscopic Retrograde Cholangiopancreatography‐Related Procedures

**DOI:** 10.1002/deo2.70333

**Published:** 2026-04-17

**Authors:** Yasuhito Kunogi, Atsushi Irisawa, Akira Yamamiya, Takumi Maki, Koh Fukushi, Ken Kashima, Fumi Sakuma, Shogo Yamamoto, Yasunori Inaba, Ryuichi Maki, Tomoya Sakamoto, Manabu Ishikawa, Ken Ohike, Keiichi Tominaga

**Affiliations:** ^1^ Department of Gastroenterology Dokkyo Medical University School of Medicine Mibu Japan

**Keywords:** balloon enteroscopy, cannulation, endoscopic sphincterotomy, ERCP, Roux‐en‐Y anastomosis

## Abstract

**Objectives:**

We aimed to evaluate the capability of a novel all‐in‐one sphincterotome in various procedures related to endoscopic retrograde cholangiopancreatography (ERCP), including procedures in patients with surgically altered anatomy.

**Methods:**

A total of 108 patients who underwent ERCP using the novel all‐in‐one sphincterotome (ENGETSU; Kaneka Medix, Tokyo, Japan) between November 2023 and December 2024 were included in this retrospective analysis. We evaluated the success rates of bile duct cannulation and endoscopic sphincterotomy (EST) as well as the incidence of procedure‐related adverse events (AEs).

**Results:**

Bile duct cannulation was performed in patients with native papillae, including 30 patients with normal anatomy, three with Billroth I reconstruction, one with Billroth II reconstruction, and six with Roux‐en‐Y reconstruction. The overall cannulation success rate was 72.5%, with a median cannulation time of 3 min. EST was performed in 65 patients with normal anatomy, three with Billroth I reconstruction, and 13 with Roux‐en‐Y reconstruction, with an overall success rate of 98.8%. Procedure‐related AEs were observed in four patients with post‐ERCP pancreatitis, three with post‐EST bleeding, and one with cholangitis; all patients recovered and were discharged. Among 14 patients in whom deep biliary cannulation failed when using the conventional ERCP cannula, subsequent use of the novel device during second‐line cannulation led to successful cannulation in 13 patients (92.9%).

**Conclusion:**

The novel all‐in‐one sphincterotome demonstrated the capability to perform various ERCP‐related procedures, including in patients with surgically altered anatomy.

**Trial Registration:**

UMIN000058337.

## 1 Introduction

**FIGURE 1:**
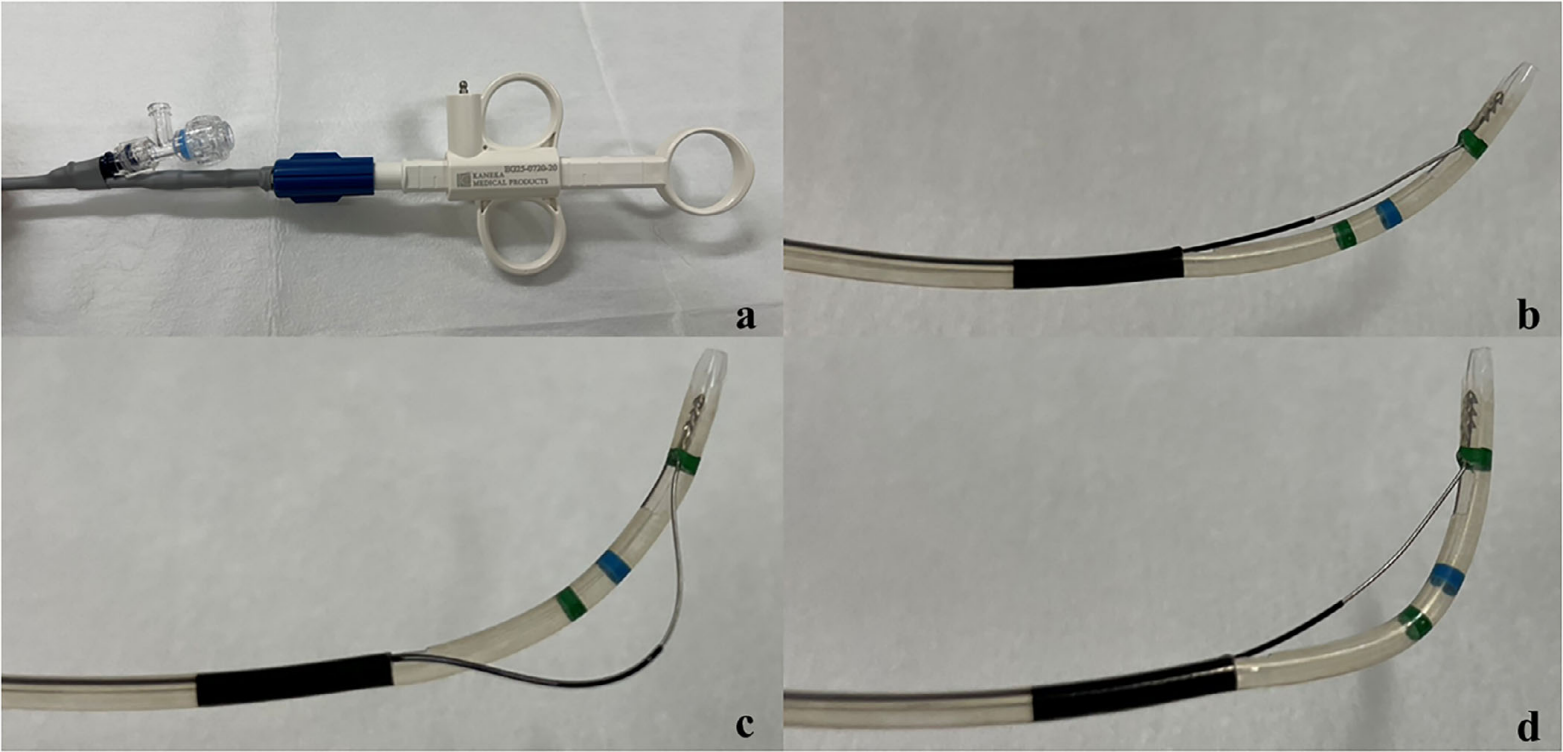
Handle (a) and tip configurations of the ENGETSU, including the standard (b), push (c), and pull (d) shapes. Reproduced from Kunogi et al., DEN Open 2024.

**FIGURE 2:**
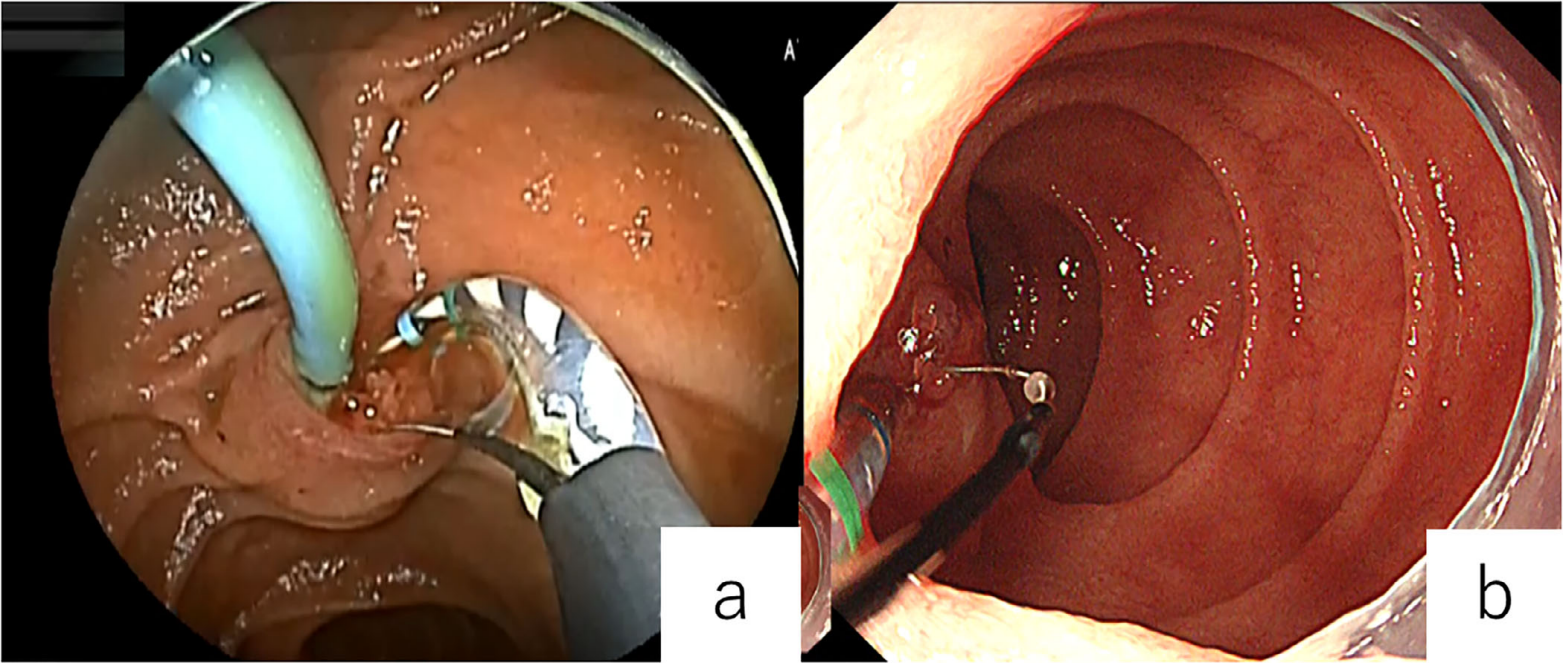
(a) Endoscopic sphincterotomy (EST) performed using the pull configuration in a Roux‐en‐Y reconstruction. (b) EST performed using the push configuration in a Roux‐en‐Y reconstruction.

The techniques and devices used in endoscopic retrograde cholangiopancreatography (ERCP) procedures have advanced significantly in recent years. However, ERCP in patients with surgically reconstructed intestinal tracts [1], which are increasingly common, as well as in those with periampullary diverticulum (PAD) [2] or postoperative anatomical alterations causing duodenal axis deviation, can often present challenges in deep biliary cannulation (DBC) and endoscopic sphincterotomy (EST). To address these difficulties, various devices and techniques have been developed, including catheters with tip‐bending functions [3], double‐lumen catheters used for the pancreatic duct guidewire method, and others [4, 5]. The novel sphincterotome (ENGETSU; Kaneka Medix, Tokyo, Japan), co‐developed by our team and Kaneka Medix, was released in May 2024. This sphincterotome features excellent rotational control and a highly flexible, freely bendable tip that allows precise manipulation of the blade's tension and deflection (Figures 1 and 2). It is expected to facilitate DBC and EST in all intestinal anatomies, including reconstructed tracts. We have previously reported the usefulness of this device in patients with reconstructed intestinal tracts [6]; however, since its release, several studies have also described effective applications of the device in complex ERCP cases with normal anatomy [7, 8, 9, 10]. The purpose of the present study was to evaluate the capability of this novel device in various ERCP‐related procedures, such as DBC, EST, and selective intrahepatic bile duct cannulation in patients requiring ERCP.

## 2 Materials and Methods

### 2.1 Study Design

Approved by the Medical Ethics Committee of Dokkyo Medical University (R‐80‐1J), this study was conducted at Dokkyo Medical University Hospital and registered with the University Hospital Medical Information Network (UMIN) Clinical Trials Registry (UMIN000058337). Informed consent was obtained via an opt‐out method, allowing participants to decline publication through our website.

The primary endpoints were the success rate of various ERCP‐related procedures using a novel all‐in‐one sphincterotome. The secondary endpoints were the incidence of adverse events (AEs) and the success rate of cannulation as a second‐line device in cases of failed DBC using a first‐line catheter.

### 2.2 Patients

We included all ERCP‐related procedures using the novel sphincterotome during the study period. We retrospectively reviewed 108 non‐consecutive cases of ERCP‐related procedures, including DBC, EST, and use as a second‐line device, performed using ENGETSU at Dokkyo Medical University Hospital between November 2023 and December 2024. The use of the all‐in‐one sphincterotome was at the discretion of the endoscopist. The device was selected for sphincterotomy or when improved axis control was needed. No patients were excluded after disclosure of information. Cases with choledochojejunal anastomosis were included in the overall cohort of ERCP‐related procedures using this device; however, they were not included in the analyses of native papilla biliary cannulation or sphincterotomy.

### 2.3 ERCP Procedure

For ERCP, the following procedures were performed: upper gastrointestinal endoscopy (GIF‐Q260J; Olympus Co., Tokyo, Japan), double‐balloon enteroscopy (EI‐580BT; Fujifilm Co., Tokyo, Japan), and single‐balloon enteroscopy (SIF‐H290S; Olympus Co., Tokyo, Japan). The side‐viewing therapeutic duodenoscopes (JF‐260V, TJF‐260V, and TJF‐Q290V; Olympus Co., Tokyo, Japan), sphincterotome (ENGETSU; Kaneka Medix, Tokyo, Japan), and standard catheter (MTW ERCP‐catheter, filiform; MTW Endoskopie Manufaktur, Wesel, Germany) were utilized. An angle‐tip wire was used as the guidewire (VisiGlide 2; Olympus Co., Tokyo, Japan, Endoselector; Boston Scientific Japan Co., Tokyo, Japan, Fielder25; Asahi Intecc Co., Ltd., Aichi, Japan). For EST, the VIO 300s electrosurgical device was used. The DBC method was selected by the operator among the following methods: wire‐guided cannulation (WGC), contrast‐guided cannulation (CGC), and pancreatic guidewire method (PGW).

The success rate of DBC was set as the percentage of DBC using the novel all‐in‐one sphincterotome by CGC, WGC, or PGW in cannulation. Cannulation was defined as failure when, at the discretion of the endoscopist, the procedure required switching to another device or the use of techniques such as the precut method. When used as the second‐line device, success was defined as achieving DBC without replacement, and failure as requiring another device or procedure termination. Cannulation time was defined as the interval from visualization of the papilla to confirmation on the contrast image. Operation time was defined as the interval from papilla visualization to procedure completion. Device switching and initial cannulation techniques were at the operator's discretion. In this study, the incision techniques included EST and endoscopic pancreatic sphincterotomy (EPST). Hereafter, EST denotes EST in the narrow sense, and the combined procedures of EST and EPST are referred to as sphincterotomy. The success rate of sphincterotomy is defined as the percentage of sphincterotomy completion using the novel all‐in‐one sphincterotome. Clinical success was defined as achieving the therapeutic goal without repeat ERCP during hospitalization. Procedures were performed by seven experts (>500 ERCPs) and eight trainees (<200 ERCPs) [11].

### 2.4 Novel All‐in‐One Sphincterotome

The novel sphincterotome [6] has a tip length of 7 mm, a knife length of 20–25 mm, a maximum diameter of the insertion part of 7.6 Fr, an effective length of 2000 mm, and a compatible guidewire diameter of 0.025 inches. It can also be used for ERCP under balloon endoscopy. Its high blade rotation and flexible tip allow incisions in both push and pull positions—the blade being semicircular in push and similar to a conventional knife in pull (Video S1).

### 2.5 Definitions of AEs

AEs were classified according to the criteria proposed by Cotton et al. [12] with reference to our previous studies [6, 12]. The severity was classified as mild, moderate, or severe, as shown in the supplementary table (Table S1).

#### 2.5.1 Bleeding

The diagnosis of hemorrhage was based on clinical evidence rather than endoscopic findings alone.

#### 2.5.2 Pancreatitis

Pancreatitis was defined as abdominal pain lasting for at least 24 h accompanied by a serum amylase level exceeding three times the upper normal limit.

#### 2.5.3 Cholangitis

Cholangitis was characterized as a septic illness lasting >24 h in an obstructed patient without any other clear source of infection.

#### 2.5.4 Others

Other AEs were classified based on the length of hospital stay and the need for surgical intervention.

#### 2.5.5 Mortality

Mortality was defined as death within 30 days directly attributable to ERCP‐related AEs involving an organ treated or traversed during the procedure.

### 2.6 Definitions of Emergency and Elective Cases

Emergency cases were defined as symptomatic cases in which ERCP was performed within 24–48 h after admission, whereas elective cases were defined as asymptomatic cases that did not require emergency ERCP.

### 2.7 Statistical Analysis

Statistical analyses were performed using SPSS version 30.0 (International Business Machines Corporation, Armonk, New York, USA). Differences in cannulation success rates among the cannulation methods were analyzed using Fisher's exact test. Comparisons between the normal anatomy group and the surgically altered anatomy group were performed using the *χ* ^2^ test or Fisher's exact test. For comparisons of biliary cannulation time, the Mann–Whitney U test was used. A *p*‐value of ≤0.05 was considered statistically significant.

## 3 Results

A total of 108 patients were analyzed in this study, with a median age of 73 years. The target diseases included common bile duct stones in 47 patients, pancreatic cancer in 14, distal bile duct cancer in eight, hilar bile duct cancer in eight, gallstones in five, intraductal papillary mucinous neoplasm in four, gallbladder cancer in three, and other diseases in 14 patients. The most common indication for ERCP was acute cholangitis. Normal anatomy was observed in 79 patients, whereas surgically altered anatomy was present in 29 patients. The latter included Roux‐en‐Y reconstruction in 18 patients, Billroth I reconstruction in three patients, Billroth II reconstruction in two patients, and choledochojejunostomy in six patients. PAD was present in 20 patients. Among them, the papilla was located outside the diverticulum in 11 patients, at the margin of the diverticulum in eight patients, and inside the diverticulum in one patient. ERCP was performed by experts in 96 cases and by trainees in 12 cases (Table 1).

**TABLE 1:** Patient characteristics.

Characteristic	Value
*n*	108
Sex, male/female	68/40
Median age, years	73 (28–91)
Emergency procedure	57 (52.8)
Benign/Malignant	64/44
Trainees/Experts	12/96
Native papilla	71 (65.7)^a^
Periampullary diverticulum	20 (18.5)
Papilla located outside the diverticulum	11 (10.2)
Papilla located at the margin of the diverticulum	8 (7.4)
Papilla located inside the diverticulum	1 (1.0)
Indication for ERCP	
Acute cholangitis	35 (32.4)
Acute cholecystitis	7 (6.5)
Obstructive jaundice	23 (21.3)
Stone removal	17 (15.7)
Detailed examination	20 (18.5)
Stent placement	6 (5.6)
Surgical reconstruction	
Normal anatomy	79 (73.1)
Billroth‐I gastrectomy	3 (2.8)
Billroth‐II gastrectomy	2 (1.9)
Roux‐en‐Y gastrectomy	18 (16.7)
Choledochojejunostomy	6 (5.6)
Target diseases	
Common bile duct stones	47
Pancreatic cancer	14
Distal bile duct cancer	8
Hilar bile duct cancer	8
Gallstones	5
Intraductal papillary mucinous neoplasm	4
Gallbladder cancer	3
Others	14
Indications for use of the novel sphincterotome^b^	
Cannulation in native papilla^a^	40
Sphincterotomy	81
Second‐line cannulation device	14
Seeking bile duct branches	6

Values are median (range) or *n* (%).

Abbreviation: ERCP, endoscopic retrograde cholangiopancreatography.

^a^ Cannulation was performed in 40 of the 71 cases.

^b^ There is overlap in the indications.

Cannulation for a native papilla was performed using the novel sphincterotome in 40 of 71 native papilla cases, including 30 with normal anatomy and 10 with surgically altered anatomy. Sphincterotomy was performed in 81 cases, of which 65 were in patients with normal anatomy, and 16 were in patients with surgically altered anatomy.

The cannulation success rate was 73.3% in patients with normal anatomy. Among patients with surgically altered anatomy, the success rate was 100% in those with Billroth I and Billroth II reconstructions, whereas it was 50% in those with Roux‐en‐Y reconstruction. Techniques included WGC, CGC, and PGW methods. Fisher's exact test showed no significant differences: WGC vs. CGC (*p* = 0.617), CGC vs. PGW (*p* = 0.422), and WGC vs. PGW (*p* = 0.172). In patients with PAD, DBC was performed in two normal‐anatomy cases with the papilla outside the diverticulum, with 100% success. When the papilla was at the diverticular margin, DBC was performed in two normal‐anatomy cases and one Roux‐en‐Y case, and was successful in all. The median cannulation time was 3 min overall, 3.5 min in patients with normal anatomy, and 6 min in patients with surgically altered anatomy. In cannulation cases, AEs consisted of one case of post‐EST bleeding and three cases of post‐ERCP pancreatitis. There were no significant differences between the normal anatomy and surgically altered anatomy groups in terms of cannulation success rate, cannulation time, incidence of AEs, or clinical success rate (Table 2).

**TABLE 2:** Results of cannulation in native papilla cases.

Characteristic	Overall	Cases of normal anatomy	Cases of surgically altered anatomy (B‐I, B‐II, R‐Y)
*n*	40	30	10
Sex, male/female	25/15	18/12	7/3
Median age, years	72.5 (28–91)	71 (28–91)	77 (49–89)
Emergency procedure	23(57.5)	15(50)	8(80)
Benign/Malignant	26/14	17/13	1/9
Trainees/Experts	2/38	2/28	0/10
Indication for ERCP			
Acute cholangitis	18 (45.0)	10 (33.3)	8 (80)
Acute cholecystitis	3 (7.5)	3 (10)	0 (0)
Obstructive jaundice	7 (17.5)	7 (23.3)	0 (0)
Stone removal	4 (10.0)	2 (6.7)	2 (20)
Detailed examination	7 (17.5)	7 (23.3)	0 (0)
Stent placement	1 (2.5)	1 (3.3)	0 (0)
Cannulation success rate	29 (72.5)	22 (73.3)	7 (70) ^*^ *p* = 0.568
Normal anatomy	22 (73.3)	22 (73.3)	n/a
Billroth‐I gastrectomy	3 (100)	n/a	3 (100)
Billroth‐II gastrectomy	1 (100)	n/a	1 (100)
Roux‐en‐Y gastrectomy	3 (50)	n/a	3 (50)
Without Periampullary diverticulum	24 (70.6)	18 (72.0)	6 (66.7)
Papilla located outside the diverticulum	2 (100)	2 (100)	n/a
Papilla located at the margin of the diverticulum	3 (100)	2 (100)	1 (100)
Papilla located inside the diverticulum	0 (0)	0 (0)	n/a
Cannulation time, min	3 (1–34)	3.5 (1–34)	6 (1–40) ^*^ *p* = 0.937
Adverse events	4 (10)	2 (6.7)	2 (20) ^*^ *p* = 0.256
Post‐EST bleeding	1 (2.5)	0 (0)	1 (10) ^*^ *p* = 0.250
Post‐ERCP pancreatitis	3 (7.5)	2 (6.7)	1 (10) ^*^ *p* = 0.589
Clinical Success	37 (92.5)	28 (93.3)	9 (90) ^*^ *p* = 0.589

Values are median (range) or *n* (%).

Abbreviations: B‐I, Billroth‐I; B‐II, Billroth‐II; EPST, endoscopic pancreatic sphincterotomy; ERCP, endoscopic retrograde cholangiopancreatography; EST, endoscopic sphincterotomy; n/a, not applicable; R‐Y, Roux‐en‐Y.

* *p*‐Value (normal anatomy vs. surgically altered anatomy).

Sphincterotomy was performed in 81 patients. Excluding one patient in whom the cutting wire broke due to contact between the wire and the endoscope, sphincterotomy was successfully completed in 80 patients, resulting in an overall success rate of 98.8%. The success rate of sphincterotomy was 98.5% in patients with normal anatomy and 100% in patients with surgically altered anatomy. Sphincterotomy success was 90% when the papilla was located outside the diverticulum, with one failure in a patient with normal anatomy. Success was 100% when the papilla was at the diverticular margin and 100% when located inside the diverticulum. One patient with surgically altered anatomy was included in both the outside‐diverticulum and margin‐diverticulum groups, and sphincterotomy was successful in both cases. In sphincterotomy cases, procedure‐related AEs occurred in seven patients (8.6%), consisting of post‐EST bleeding in three, post‐ERCP pancreatitis in three, and post‐ERCP cholangitis in one patient; all patients recovered and were discharged. The clinical success rate was 91.4%. There were no significant differences between the normal anatomy and surgically altered anatomy groups in terms of sphincterotomy success rate, incidence of AEs, or clinical success rate (Table 3).

**TABLE 3:** Results of sphincterotomy (including endoscopic sphincterotomy [EST] and endoscopic pancreatic sphincterotomy [EPST]) cases.

Characteristic	Overall	Cases of normal anatomy	Cases of surgically altered anatomy (B‐I, R‐Y)
*n*	81	65	16
Sex, male/female	50/31	39/26	11/5
Median age, years	74.0 (28–91)	73 (28–91)	77 (45–89)
Emergency procedure	38 (46.9)	31 (47.7)	7 (43.8)
Benign/Malignant	45/36	33/32	12/4
Papilla without EST or EPBD	77	61	16
Indication for sphincterotomy			
Stent placement	49 (60.5)	43 (66.2)	6 (37.5)
Stone removal	27 (33.3)	17 (26.2)	10 (62.5)
Others	5 (6.2)	5 (7.7)	0 (0)
Sphincterotomy success rate	80 (98.8)	64 (98.5)	16 (100) ^*^ *p* = 0.802
Normal anatomy	64 (98.5)	64 (98.5)	n/a
Billroth‐I gastrectomy	3 (100)	n/a	3 (100)
Roux‐en‐Y gastrectomy	13 (100)	n/a	13 (100)
Without Periampullary diverticulum	64 (100)	50 (100)	14 (100)
Papilla located outside the diverticulum	9 (90)	8 (88.9)	1 (100)
Papilla located at the margin of the diverticulum	6 (100)	5 (100)	1 (100)
Papilla located inside the diverticulum	1 (100)	1 (100)	n/a
Adverse events	7 (8.6)	4 (6.2)	3 (18.7) ^*^ *p* = 0.135
Post‐EST hemorrhage	3 (3.7)	2 (3.1)	1 (6.3) ^*^ *p* = 0.448
Post‐ERCP pancreatitis	3 (3.7)	1 (1.5)	2 (12.5) ^*^ *p* = 0.098
Cholangitis	1 (1.2)	1 (1.5)	0 (0) ^*^ *p* = 0.802
Clinical Success	74 (91.4)	59 (90.8)	15 (93.8) ^*^ *p* = 0.580

Values are median (range) or *n* (%).

Abbreviations: B‐I, Billroth‐I; B‐II, Billroth‐II; ERCP, endoscopic retrograde cholangiopancreatography; EST, endoscopic sphincterotomy; n/a, not applicable; R‐Y, Roux‐en‐Y.

* *p*‐Value (normal anatomy vs. surgically altered anatomy).

The all‐in‐one sphincterotome was used as a second‐line device for cannulation in 14 cases in which the initial device failed. The initial device in all cases was a conventional ERCP cannula. Among these, seven cases involved native papillae and seven involved previously treated papillae. Overall, successful cannulation was achieved in 13 of 14 cases (92.9%). The success rates by anatomy were 87.5% (7/8) for normal anatomy, 100% (1/1) for Billroth II, and 100% (5/5) for Roux‐en‐Y reconstruction. Results were also analyzed separately for untreated and treated papillae. In the untreated papilla group, four cases had normal anatomy, and three had Roux‐en‐Y reconstruction, and cannulation was successful in all seven cases (100%). In the treated papilla group, there were four cases with normal anatomy, one with Billroth II reconstruction, and two with Roux‐en‐Y reconstruction. Cannulation was successful in six of seven cases (85.7%) overall, with success rates of 75% (3/4) for normal anatomy and 100% for both Billroth II and Roux‐en‐Y reconstructions (Table 4).

**TABLE 4:** Cases of cannulation using all‐in‐one sphincterotome as a second‐line device.

Age	Sex	Disease	Reconstruction method	First‐line device	Cannulation method^a^	Overall cannulation time(minutes)	Cannulation success	Clinical success
73	Male	Bile duct stricture from renal cyst compression	Normal anatomy	EC	WGC, **Precut**	30	Yes	Yes
66	Male	BSALT	Normal anatomy	EC	WGC, **WGC**	5	Yes	Yes
77	Female	Pancreatic cancer	Normal anatomy	EC	CGC, WGC, **WGC**	30	Yes	Yes
69	Female	ICC	Normal anatomy	EC	WGC, **WGC**	15	Yes	Yes
68	Female	CBDS	Normal anatomy	EC	WGC, **CGC**	31	Yes	Yes
80	Male	Pancreatic leakage	Normal anatomy	EC	WGC, **WGC**	25	Yes	No
68	Male	Chronic pancreatitis	Normal anatomy	EC	WGC, **WGC**	16	Yes	Yes
56	Male	Hilar bile duct cancer	Normal anatomy	EC	WGC, **PGW**, **Precut**, WGC	25	No	Yes
68	Female	HCC	B‐II	EC	WGC, **CGC**	11	Yes	Yes
67	Male	DC	R‐Y	EC	WGC, **WGC**	40	Yes	Yes
75	Male	CBDS	R‐Y	EC	CGC, WGC, **WGC**	19	Yes	Yes
73	Male	CBDS	R‐Y	EC	WGC, **CGC**	22	Yes	Yes
84	Male	CBDS	R‐Y	EC	WGC, through‐the‐stent, **WGC**	52	Yes	Yes
83	Male	CBDS	R‐Y	EC	WGC, **WGC**	23	Yes	Yes

Abbreviations: B‐II, Billroth‐II; BSALT, biliary stricture after liver transplantation; CBDS, common bile duct stone; CGC, contrast guided cannulation; DC, distal cholangiocarcinoma; EC, standard ERCP catheter; HCC, hepatocellular carcinoma; ICC, intrahepatic cholangiocarcinoma; PGW, pancreatic guide‐wire; R‐Y, Roux‐en‐Y; WGC, wire guided cannulation.

^a^ The procedures are described in the order in which they were performed. When cannulation was successfully achieved using the all‐in‐one sphincterotome as a second‐line device, the corresponding technique is indicated in bold.

In addition, the novel all‐in‐one sphincterotome was used for selective cannulation of intrahepatic bile duct branches in five cases.

## 4 Discussion

We collaborated with Kaneka Medix to develop ENGETSU, which was commercialized in May 2024.

ENGETSU features two key innovations: superior rotational performance, enabling precise transmission of handle rotation to the distal tip, and exceptional flexibility with free bow‐up and bow‐down movements. These features improve control of papilla approach and incision direction, enhancing the success of cannulation and EST in difficult cases. DBC of native papillae was performed in 40 cases, with an overall success rate of 72.5%. Cannulation was successful in 73.3% of patients with normal anatomy and in all Billroth I and II cases, whereas the rate was 50.0% in Roux‐en‐Y cases, likely reflecting early pilot experience and the definition of failure at device switch. Favorable results when ENGETSU was used as a second‐line device in Roux‐en‐Y cases suggest improved performance with increased experience. Cannulation outcomes in other anatomical settings were comparable to those reported with conventional sphincterotomes [13]. Although the number of cases was limited, successful cannulation was also achieved in patients with PAD, indicating that ENGETSU has the capability to manage anatomically challenging situations. Although PAD is associated with difficult cannulation, final success rates are comparable with appropriate techniques [14, 15]. In the present study, cannulation was achieved without device exchange in all but one case involving a papilla located inside the diverticulum. Although precutting in PAD cases requires advanced technical skill, the flexible distal tip and superior rotational performance of this device facilitated axis alignment and enabled cannulation without resorting to adjunctive techniques. These findings suggest its utility in PAD cases.

Sphincterotomy was performed in 81 patients, with successful completion achieved in 80 cases, yielding an overall success rate of 98.8%. High sphincterotomy and clinical success rates were achieved across all anatomical types, including 65 patients with normal anatomy, three with Billroth I reconstruction, and 13 with Roux‐en‐Y reconstruction. In addition, EST was successfully completed in all patients with PAD located at the diverticular margin in six cases and inside the diverticulum in one case. Sphincterotomy in patients with reconstructed intestinal tracts is regarded as a technically challenging procedure [16]. The present findings demonstrated the high utility of ENGETSU, particularly in such reconstructed cases. Given these results, using ENGETSU as the first‐choice device in ERCP procedures requiring sphincterotomy may help reduce procedure time and overall cost.

In contrast, ENGETSU was used as a second‐line device in 14 cases after unsuccessful cannulation with a conventional catheter, achieving successful cannulation in 13 cases (92.9%). According to anatomical classification, the success rates were 87.5% (7/8) in patients with normal anatomy, 100% (1/1) in Billroth II reconstruction, and 100% (5/5) in Roux‐en‐Y reconstruction, demonstrating particularly high success rates in patients with surgically altered anatomy. For difficult cannulation, salvage techniques such as the rendezvous method [17, 18] and precut technique [19] have been reported, with success rates of approximately 82% and 77%–100% [20], respectively. These techniques require expertise and may increase AEs [17]. In contrast, salvage cannulation with an all‐in‐one sphincterotome requires only catheter exchange. By adjusting the axis through controlled rotation and flexion of the distal tip, cannulation was achieved without resorting to precut. In the present study, ENGETSU demonstrated a high success rate as a second‐line device, suggesting that it may serve as a less invasive alternative to conventional salvage techniques in difficult ERCP cases. Adverse events occurred in eight of 108 patients: post‐EST bleeding in three, post‐ERCP pancreatitis in four, and cholangitis in one. All recovered with conservative treatment. The incidence of AEs was comparable to current guidelines and previous reports [16, 21, 22], indicating an acceptable safety profile for this device.

ERCP is widely used for diagnosing and treating biliary and pancreatic diseases, with DBC being a key determinant of success. However, cannulation and sphincterotomy can be technically challenging in cases with PAD, duodenal axis deviation, or surgically altered anatomy, where the papilla is often tangentially oriented. In reconstructed anatomy, sphincterotomy is typically approached from the anal side, making the standard oral‐side incision difficult. Techniques such as precutting after plastic stent placement [23] have been proposed, but they require considerable expertise. Alternative approaches, including endoscopic ultrasound–guided biliary drainage, are also available; however, these are limited to specialized centers and carry risks such as fistula‐related complications and stent migration. The present findings suggest that, owing to its superior rotational performance and flexible distal tip, ENGETSU has the capability to manage these anatomically challenging situations. Because this study did not include a comparison with other sphincterotomes, the present findings should be interpreted as descriptive data demonstrating the capability of this device rather than evidence of its clinical superiority.

This study has some limitations. First, it was a single‐center, retrospective study. Second, most of the procedures were performed by experts. Therefore, further studies are needed to determine whether ENGETSU is useful as a training tool for trainees. In addition, as this study did not include a comparison with sphincterotomes produced by other manufacturers, randomized controlled trials and other prospective studies will be necessary to objectively evaluate the superiority of ENGETSU.

## 5 Conclusion

The novel all‐in‐one sphincterotome (ENGETSU) demonstrated good performance in both sphincterotomy and DBC, including in patients with surgically altered anatomy and other difficult cases. Its superior tip flexibility and high rotational control allowed precise and effective procedures, even as a second‐line device after initial failure. ENGETSU may be a promising tool that has the potential to improve technical success rates in challenging ERCP cases.

## Author Contributions

Yasuito Kunogi and Akira Yamamiya designed this manuscript, collected and analyzed the data, and drafted the manuscript; Akira Yamamiya and Atsushi Irisawa checked the manuscript and approved the final version; Ken Ohike, Manabu Ishikawa, Tomoya Sakamoto, Ryuichi Maki, Yaunori Inaba, Ken Kashima, Fumi Sakuma, Koh Fukushi, Shogo Yamamoto, and Takumi Maki collected the data and created the tables; Keiichi Tominaga and Atsushi Irisawa supervised the preparation of this manuscript.

## Funding

The authors have nothing to report.

## Ethics Statement

This retrospective study was conducted in accordance with the Declaration of Helsinki and was approved by the Medical Ethics Committee of Dokkyo Medical University (approval number: R‐80‐1J).

## Consent

Because of the retrospective nature of the study, informed consent was obtained using an opt‐out method, allowing patients to refuse participation through information disclosed on the institutional website.

## Conflicts of Interest

The authors declare no conflicts of interest.

## Supporting information




**Supporting Table 1**: Severity classification of adverse events associated with ERCP according to the Cotton criteria.

**Supporting Video 1**: This video demonstrates the rotational mechanism of the novel all‐in‐one sphincterotome, endoscopic sphincterotomy (EST) performed in patients with normal intestinal anatomy, and EST performed in patients with surgically reconstructed intestinal anatomy using the pull‐type and push‐type knife positions.
