# Supplementary material for: The Capability of a Novel All‐in‐One Sphincterotome With High Rotation and a Freely Bendable Blade for Various Endoscopic Retrograde Cholangiopancreatography‐Related Procedures
Source: DEN Open. 2026 Apr 17;6(1):e70333. doi: 10.1002/deo2.70333 (PMC13088330; doi:10.1002/deo2.70333)
Supplement: Supplementary file 1 — Supporting Table 1: Severity classification of adverse events associated with ERCP according to the Cotton criteria. [file DEO2-6-e70333-s002.docx]

Supplementary Table 1　　Severity Classification of Adverse Events in ERCP

|  | Mild | Moderate | Severe |
| --- | --- | --- | --- |
| Bleeding | Hemoglobin decrease of ≤3 g/dL without the need for blood transfusion | Hemorrhage requiring transfusion of up to four units of blood without the need for angiographic or surgical intervention | Hemorrhage requiring transfusion of five or more units of blood or requiring angiographic or surgical intervention |
| Pancreatitis | Hospitalization for up to 3 days | Prolonged hospitalization for 4–10 days | Hospitalization for more than 10 days, or development of complications such as phlegmon, pseudocyst, or hemorrhage, or the need for additional interventional treatment |
| Choangitis(Infection) | Symptoms lasting <48 hours | Symptoms lasting ≥3 days requiring hospital treatment, or requiring endoscopic or percutaneous intervention | Septic shock or requiring emergent surgical intervention |
| Others | Prolonged hospitalization for up to 3 days | Prolonged hospitalization for 4–10 days | Prolonged hospitalization exceeding 10 days or requiring intensive care or surgical treatment |
